# Supplementary material for: Adverse events of special interest and mortality following vaccination with mRNA (BNT162b2) and inactivated (CoronaVac) SARS-CoV-2 vaccines in Hong Kong: A retrospective study
Source: PLoS Med. 2022 Jun 21;19(6):e1004018. doi: 10.1371/journal.pmed.1004018 (PMC9212142; doi:10.1371/journal.pmed.1004018)
Supplement: S1 Table — (PDF) [file pmed.1004018.s002.pdf]

S1 Table. Definitions of adverse events of special interest (AESI)

AESI	ICD-9-CM diagnosis and procedure codes, and laboratory parameters
Guillain-Barré Syndrome	357.0 Acute infective polyneuritis<br>357.8x Other inflammatory and toxic neuropathy<br>357.9 Inflammatory/toxic neuropathy, unspecified
Acute disseminated encephalomyelitis (ADEM)	323.6x Postinfectious encephalitis, myelitis, and encephalomyelitis<br>323.8x Other causes of encephalitis, myelitis and encephalomyelitis
Sleeping disturbance or disorder	347.xx Cataplexy and narcolepsy<br>307.4 (Nonorganic sleep disorder, unspecified),<br>780.5 (unspecified sleep disturbance)<br>[Procedure codes:<br>89.17 Polysomnography<br>89.18 Multiple sleep latency]
Acute aseptic arthritis	274.0x Gouty arthropathy<br>696.0 Psoriatic arthropathy<br>716.5x Unspecified polyarthropathy or polyarthritis<br>716.6x Unspecified monoarthritis<br>716.9x Unspecified arthropathy<br>712.xx Crystal arthropathies<br>711.5x Arthropathy associated with other viral diseases
Type 1 Diabetes	250.01 Diabetes mellitus without mention of complication, type I [juvenile type], not stated as uncontrolled<br>250.03 Diabetes mellitus without mention of complication, type I [juvenile type], uncontrolled<br>250.11 Diabetes with ketoacidosis, type I [juvenile], not stated as uncontrolled<br>250.13 Diabetes with ketoacidosis, type I [juvenile], uncontrolled<br>250.21 Diabetes with hyperosmolarity, type I [juvenile], not stated as uncontrolled<br>250.23 Diabetes with hyperosmolarity, type I [juvenile], uncontrolled<br>250.31 Diabetes with other coma, type I [juvenile], not stated as uncontrolled<br>250.33 Diabetes with other coma, type I [juvenile], uncontrolled<br>250.41 Diabetes with renal manifestations, type I [juvenile], not stated as uncontrolled<br>250.43 Diabetes with renal manifestations, type I [juvenile], uncontrolled<br>250.51 Diabetes with ophthalmic manifestations, type I [juvenile], not stated as uncontrolled<br>250.53 Diabetes with ophthalmic manifestations, type I [juvenile], uncontrolled<br>250.61 Diabetes with neurological manifestations, type I [juvenile], not stated as uncontrolled<br>250.63 Diabetes with neurological manifestations, type I [juvenile], uncontrolled<br>250.71 Diabetes with peripheral circulatory disorders, type I [juvenile], not stated as uncontrolled<br>250.73 Diabetes with peripheral circulatory disorders, type I [juvenile], uncontrolled<br>250.81 Diabetes with other specified manifestations, type I [juvenile], not stated as uncontrolled<br>250.83 Diabetes with other specified manifestations, type I [juvenile], uncontrolled 250.91 Diabetes with unspecified complications, type I [juvenile], not stated as uncontrolled<br>250.93 Diabetes with unspecified complications, type I [juvenile], uncontrolled

(Idiopathic) Thrombocytopenia	287.3x Primary thrombocytopenia<br>287.4x Secondary thrombocytopenia<br>287.5 Thrombocytopenia, unspecified<br>279.1x Deficiency of cell-mediated immunity<br>283.1x Non-autoimmune hemolytic anemias<br>284.1x Drug-induced bone marrow suppression<br>446.6 Thrombotic microangiopathy<br>776.1 Transient neonatal thrombocytopia
Subacute thyroiditis	245.1 Subacute thyroiditis
Acute cardiovascular injury including:	
Microangiopathy	446.6 Thrombotic microangiopathy
Heart failure	428.x Heart failure<br>398.9x Other and unspecified rheumatic heart diseases<br>402.xx Hypertensive heart disease (with or without heart failure)<br>404.xx Hypertensive heart and chronic kidney disease (with or without heart failure)
Stress cardiomyopathy	429.8x Other ill-defined heart diseases
Arrhythmia	427.xx Cardiac dysrhythmias<br>426.7 Anomalous atrioventricular excitation<br>794.3x Nonspecific abnormal results of function study of cardiovascular system<br>785.0 Tachycardia, unspecified
Carditis	420.9 Pericarditis<br>422.xx Acute myocarditis<br>423.9 Unspecified disease of pericardium<br>429.0 Myocarditis, unspecified
Thromboembolism<br>(DVT, PE, stroke, limb ischemia)	410.x Acute myocardial infraction<br>415.1x Pulmonary embolism and infarction<br>453.x Other venous embolism and thrombosis<br>443.x Other peripheral vascular disease<br>444.x Arterial embolism and thrombosis<br>445.x Atheroembolism<br>433.x Occlusion and stenosis of precerebral arteries<br>434.x Occlusion of cerebral arteries<br>435.x Transient cerebral ischemia (TIA)<br>436.x CVA<br>437.x Other and ill-defined cerebrovascular disease<br>451.x Phlebitis and thrombophlebitis<br>452.x Portal vein thrombosis<br>453.40 Acute venous embolism and thrombosis of unspecified deep vessels of lower extremity<br>453.41 Acute venous embolism and thrombosis of deep vessels of proximal lower extremity<br>453.42 Acute venous embolism and thrombosis of deep vessels of distal lower extremity<br>459.9 Unspecified circulatory system disorder<br>325.x Phlebitis and thrombophlebitis and intracranial venous sinuses<br>286.6x Defibrination syndrome

	557.0 Acute vascular insufficiency of intestine<br>557.9 Unspecified vascular insufficiency of intestine<br>[Procedure codes:<br>36.0x Removal Of Coronary Artery Obstruction And Insertion Of Stent(s)<br>36.1x Bypass Anastomosis For Heart Revascularization<br>36.2x heart revascularization by arterial implant<br>36.3x other heart revascularization]
Coronary artery disease	410.x-414.x Ischemic Heart Disease<br>V45.81 Presence of aortocoronary bypass graft<br>[Procedure codes:<br>36.0x Removal Of Coronary Artery Obstruction And Insertion Of Stent(s)<br>36.1x Bypass Anastomosis For Heart Revascularization<br>36.2x heart revascularization by arterial implant<br>36.3x other heart revascularization]
Myocardial Infarction	410.x-414.x Ischemic Heart Disease<br>V45.81 Presence of aortocoronary bypass graft<br>[Procedure codes:<br>36.0x Removal Of Coronary Artery Obstruction And Insertion Of Stent(s)<br>36.1x Bypass Anastomosis For Heart Revascularization<br>36.2x heart revascularization by arterial implant<br>36.3x other heart revascularization]
Venous thromboembolism	415.1x Pulmonary embolism and infarction<br>453.xx Other venous embolism and thrombosis
Arterial thromboembolism	433.xx Occlusion and stenosis of precerebral arteries<br>434.xx Occlusion of cerebral arteries<br>435.x Transient cerebral ischemia<br>436 Acute but ill-defined cerebrovascular disease<br>437.0 Cerebral atherosclerosis<br>437.1 Other generalized ischemic cerebrovascular disease<br>437.6 Nonpyogenic thrombosis of intracranial venous sinus<br>437.7 Transient global amnesia<br>437.8 Other ill-defined cerebrovascular disease<br>437.9 Unspecified cerebrovascular disease
Hemorrhagic disease	286.5x Hemorrhagic disorder due to intrinsic circulating anticoagulants<br>286.7 Acquired coagulation factor deficiency<br>287.xx Purpura and other hemorrhagic conditions<br>430.x Subarachnoid hemorrhage<br>431.x Intracerebral hemorrhage<br>432.x Nontraumatic extradural hemorrhage<br>[Procedure codes:<br>99.06 Transfusion of coagulation factors]

Single Organ Cutaneous Vasculitis	709.1 Vascular disorders of skin<br>446.2x Hypersensitivity angiitis<br>287.0 Allergic purpura
Acute liver injury	570.x Liver acute (subacute)<br>573.3 (Hepatitis, toxic)
Acute kidney injury	584.x Acute kidney failure<br>586.xx Renal failure unspecified<br>[Lab parameters: ≥1.5-fold increase in serum creatinine within 7 days or ≥ 26.5 µmol/L absolute increase in serum creatinine within 2 days]
Acute pancreatitis	577.0 Acute pancreatitis
Generalized convulsion	345.xx Epilepsy and recurrent seizures<br>780.3x Convulsions<br>779.0 Convulsions in newborn<br>333.2 Myoclonus<br>649.4 Epilepsy complicating pregnancy, childbirth, or the puerperium
Meningoencephalitis	322.9x Meningitis, unspecified<br>323.0x Encephalitis, myelitis and encephalomyelitis in viral diseases classified elsewhere<br>323.4x Other encephalitis, myelitis and encephalomyelitis due to infections classified elsewhere<br>323.5x Encephalitis, myelitis and encephalomyelitis following immunization procedures<br>323.6x Postinfectious encephalitis, myelitis, and encephalomyelitis<br>323.8x Other causes of encephalitis, myelitis and encephalomyelitis<br>323.9x Unspecified causes of encephalitis, myelitis, and encephalomyelitis<br>323.7 Toxic encephalitis<br>330.8 Acute necrotizing hemorrhagic encephalopathy<br>377.7 Disorders of visual cortex<br>046.3 Telencephalic leukoencephalopathy<br>049.8 Rio Bravo Fever<br>036.1 Bacterial encephalitis<br>056.0 Rubella with neurological complications<br>136.2 Specific infections by free-living amebae<br>130.xx Toxoplasmosis<br>054.3 Herpetic meningoencephalitis<br>049.0 Lymphocytic choriomeningitis<br>094.1 General paresis<br>072.2 Mumps encephalitis<br>013.0 Meningoencephalitis (infectious origin)<br>062.4 Murray valley encephalitis<br>049.9 Viral encephalitis<br>045.0 Polio encephalitis<br>062.xx Mosquito-borne viral encephalitis

Transverse myelitis	323.0x Encephalitis, myelitis, and encephalomyelitis in viral diseases classified elsewhere<br>323.4x Other encephalitis, myelitis, and encephalomyelitis due to infection classified elsewhere<br>323.5x Encephalitis, myelitis, and encephalomyelitis following immunization procedures<br>323.6x Postinfectious encephalitis, myelitis, and encephalomyelitis<br>323.8x Other causes of encephalitis, myelitis, and encephalomyelitis<br>341.2x Acute (transverse) myelitis
Bell's palsy	351.0 Bell's palsy<br>351.8 (other facial nerve disorders)<br>351.9 (facial nerve disorder, unspecified)
Acute respiratory distress syndrome	518.8x Other pulmonary insufficiency, not elsewhere classified<br>518.5x Pulmonary insufficiency following trauma and surgery<br>[Procedure codes:<br>96.7x Continuous mechanical ventilation]
Erythema multiforme	695.1x Erythema multiforme
Chilblain – like lesions	991.5 Chilblains
Anosmia, ageusia	781.1 Disturbances of sensation of smell and taste
Anaphylaxis	995.0 Anaphylactic reaction, unspecified<br>995.1 Angioneurotic edema<br>995.3 Allergy, unspecified, not elsewhere classified<br>995.4 Shock due to anesthesia, not elsewhere classified<br>999.4x Anaphylactic reaction to serum<br>708.9 Urticaria<br>519.1 Other diseases of trachea and bronchus not elsewhere classified<br>786.1 Stridor<br>458.9 Hypotension
Multisystem inflammatory syndrome in children	446.1 Mucocutaneous lymph node syndrome (Kawasaki disease)
Sudden death	798.x Sudden death cause unknown<br>799 Other ill- defined and unknown causes of morbidity and mortality
Rhabdomyolysis	728.8 Other disorders of muscle ligament and fascia<br>791.3 Myoglobinuria

Abbreviations: AESI=adverse event of special interest; ICD-9-CM=International Classification of Diseases, Ninth Revision, Clinical Modification
