# Supplementary material for: Adverse events of special interest and mortality following vaccination with mRNA (BNT162b2) and inactivated (CoronaVac) SARS-CoV-2 vaccines in Hong Kong: A retrospective study
Source: PLoS Med. 2022 Jun 21;19(6):e1004018. doi: 10.1371/journal.pmed.1004018 (PMC9212142; doi:10.1371/journal.pmed.1004018)
Supplement: S2 Table — (PDF) [file pmed.1004018.s003.pdf]

S2 Table. Goodness-of-fit of Poisson regression models

AESI	First dose recipients			Second dose recipients		
	Deviance	P-value for deviance Chi-square test	P-value for overdispersion	Deviance	P-value for deviance Chi-square test	P-value for overdispersion
Overall AESI	55,626	1.000	0.636	47,079	1.000	0.660
Guillain-Barré Syndrome	-	-	-	-	-	-
Acute disseminated encephalomyelitis	-	-	-	-	-	-
Sleeping disturbance or disorder	7,858	1.000	0.624	6,716	1.000	0.654
Acute aseptic arthritis	2,739	1.000	0.929	2,800	1.000	0.912
Type 1 Diabetes	-	-	-	-	-	-
(Idiopathic) Thrombocytopenia	723	1.000	0.821	-	-	-
Subacute thyroiditis	-	-	-	-	-	-
Microangiopathy	-	-	-	-	-	-
Heart failure	3,200	1.000	0.317	2,089	1.000	0.871
Stress cardiomyopathy	-	-	-	-	-	-
Arrhythmia	10,224	1.000	0.999	8,085	1.000	0.762
Carditis	-	-	-	-	-	-
Thromboembolism	16,958	1.000	0.865	13,484	1.000	0.895
Coronary artery disease	10,364	1.000	0.586	8,979	1.000	0.319
Myocardial Infarction	7,044	1.000	0.542	5,756	1.000	0.845
Venous thromboembolism	1,213	1.000	0.808	-	-	-
Arterial thromboembolism	9,732	1.000	0.915	7,906	1.000	0.844
Hemorrhagic disease	3,904	1.000	0.864	2,962	1.000	0.629
Single Organ Cutaneous Vasculitis	-	-	-	-	-	-
Acute liver injury	1,139	1.000	0.666	919	1.000	0.840
Acute kidney injury	8,528	1.000	0.150	7,194	1.000	0.575
Acute pancreatitis	1,810	1.000	0.784	1,587	1.000	0.370
Generalized convulsion	4,757	1.000	0.351	2,859	1.000	0.963
Meningoencephalitis	-	-	-	-	-	-
Transverse myelitis	-	-	-	-	-	-
Bell’s palsy	2,819	1.000	0.928	2,638	1.000	0.711
Acute respiratory distress syndrome	1,577	1.000	0.912	2,799	1.000	0.893
Erythema multiforme	-	-	-	-	-	-
Chilblain – like lesions	-	-	-	-	-	-
Anosmia, ageusia	-	-	-	-	-	-
Anaphylaxis	1,430	1.000	0.152	-	-	-
Multisystem inflammatory syndrome in children	-	-	-	-	-	-
Sudden death	1,035	1.000	0.878	1,240	1.000	0.795
Rhabdomyolysis	680	1.000	0.561	-	-	-
All-cause mortality	4,172	1.000	0.506	3,083	1.000	0.950

Abbreviations: AESI=adverse event of special interest

Note: Goodness-of-fit of Poisson regression models were assessed only when there were at least five events for a specific AESI in both BNT162b2 and CoronaVac groups, respectively.
