# Supplementary material for: Adverse events of special interest and mortality following vaccination with mRNA (BNT162b2) and inactivated (CoronaVac) SARS-CoV-2 vaccines in Hong Kong: A retrospective study
Source: PLoS Med. 2022 Jun 21;19(6):e1004018. doi: 10.1371/journal.pmed.1004018 (PMC9212142; doi:10.1371/journal.pmed.1004018)
Supplement: S3 Table — (PDF) [file pmed.1004018.s004.pdf]

S3 Table. Sensitivity analysis of the study outcomes by excluding people with previous SARS-CoV-2 infection

AESI	Excluding COVID survivors					
	First dose recipients			Second dose recipients		
	CoronaVac vs BNT162b2 (ref)			CoronaVac vs BNT162b2 (ref)		
	IRR	95% CI	P-value	IRR	95% CI	P-value
Overall AESI	0.98	(0.89, 1.09)	0.726	0.97	(0.87, 1.08)	0.546
Sleeping disturbance or disorder	0.66	(0.49, 0.88)	0.006	0.91	(0.66, 1.27)	0.586
Acute aseptic arthritis	1.33	(0.79, 2.24)	0.282	1.13	(0.68, 1.90)	0.636
(Idiopathic) Thrombocytopenia	0.72	(0.24, 2.16)	0.556	-	-	-
Heart failure	1.59	(0.94, 2.71)	0.083	1.45	(0.77, 2.74)	0.255
Coronary artery disease	1.13	(0.88, 1.44)	0.354	1.13	(0.86, 1.47)	0.376
Arrhythmia	0.90	(0.69, 1.17)	0.432	0.96	(0.72, 1.27)	0.764
Thromboembolism	0.94	(0.78, 1.14)	0.542	0.93	(0.75, 1.15)	0.501
Hemorrhagic disease	1.04	(0.67, 1.60)	0.870	0.63	(0.38, 1.04)	0.070
Acute liver injury	1.31	(0.57, 2.98)	0.523	0.59	(0.21, 1.66)	0.317
Acute kidney injury	2.24	(0.97, 5.16)	0.059	0.79	(0.30, 2.06)	0.624
Acute pancreatitis	1.08	(0.51, 2.30)	0.838	1.62	(0.81, 3.24)	0.173
Generalized convulsion	1.41	(0.92, 2.17)	0.117	0.73	(0.43, 1.25)	0.250
Bell’s palsy	1.95	(1.12, 3.41)	0.018	0.88	(0.51, 1.51)	0.641
Acute respiratory distress syndrome	0.81	(0.40, 1.66)	0.571	1.27	(0.76, 2.13)	0.360
Anaphylaxis	0.34	(0.14, 0.79)	0.012	-	-	-
Sudden death	0.97	(0.39, 2.38)	0.943	1.80	(0.69, 4.73)	0.232
Rhabdomyolysis	1.48	(0.46, 4.79)	0.513	-	-	-
Myocardial Infarction	1.18	(0.87, 1.60)	0.287	0.91	(0.65, 1.28)	0.604
Venous thromboembolism	0.57	(0.26, 1.29)	0.180	-	-	-
Arterial thromboembolism	1.02	(0.79, 1.33)	0.858	1.12	(0.84, 1.50)	0.446
All-cause mortality	0.96	(0.63, 1.48)	0.866	0.85	(0.51, 1.40)	0.516

Abbreviations: AESI=adverse event of special interest; CI=confidence interval; IRR=incidence rate ratio

Note: IRR was estimated only when there were at least five events for a specific AESI in both BNT162b2 and CoronaVac groups, respectively.
