# Supplementary material for: Adverse events of special interest and mortality following vaccination with mRNA (BNT162b2) and inactivated (CoronaVac) SARS-CoV-2 vaccines in Hong Kong: A retrospective study
Source: PLoS Med. 2022 Jun 21;19(6):e1004018. doi: 10.1371/journal.pmed.1004018 (PMC9212142; doi:10.1371/journal.pmed.1004018)
Supplement: S1 Fig — IPTW, inverse probability of treatment weighting. (PDF) [file pmed.1004018.s005.pdf]

S1 Fig. The observed propensity score weighting distribution after truncation of <1st and >99th percentiles by the first and second vaccine dose

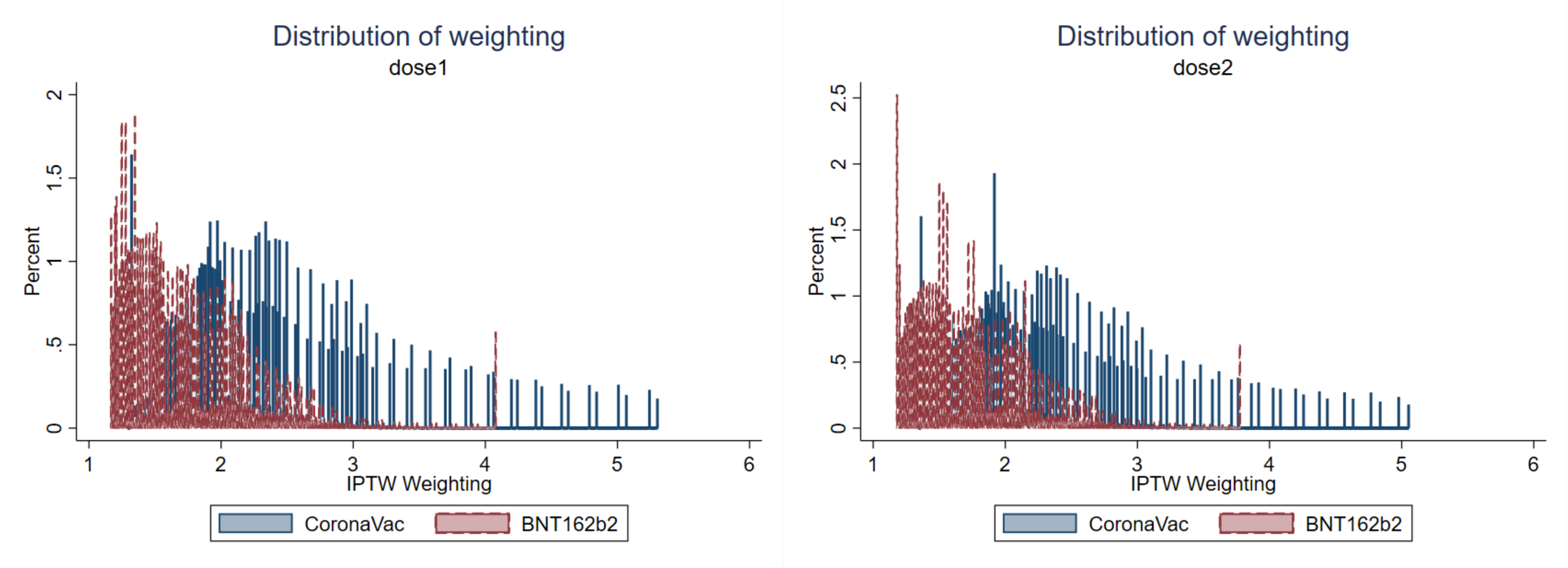

Note: IPTW=inverse probability of treatment weighting
