# Supplementary material for: Adverse events of special interest and mortality following vaccination with mRNA (BNT162b2) and inactivated (CoronaVac) SARS-CoV-2 vaccines in Hong Kong: A retrospective study
Source: PLoS Med. 2022 Jun 21;19(6):e1004018. doi: 10.1371/journal.pmed.1004018 (PMC9212142; doi:10.1371/journal.pmed.1004018)

S3 Fig. Distribution of occurrence of sleeping disturbance or disorder within 21 days after the first dose of BNT162b2

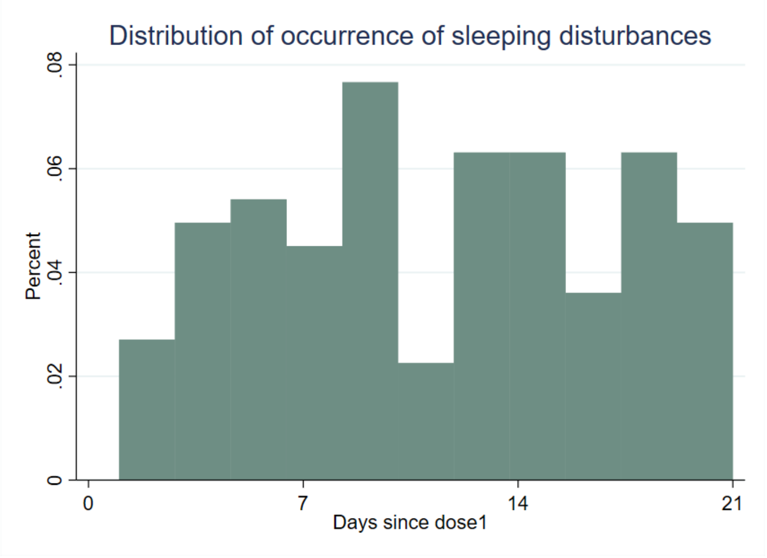

Supplement: S3 Fig — (PDF) [file pmed.1004018.s007.pdf]
