# Supplementary material for: Adverse events of special interest and mortality following vaccination with mRNA (BNT162b2) and inactivated (CoronaVac) SARS-CoV-2 vaccines in Hong Kong: A retrospective study
Source: PLoS Med. 2022 Jun 21;19(6):e1004018. doi: 10.1371/journal.pmed.1004018 (PMC9212142; doi:10.1371/journal.pmed.1004018)
Supplement: S1 Protocol — (PDF) [file pmed.1004018.s008.pdf]

S1 Protocol. Study protocol

Manuscript Title: Adverse Events of Special Interest and mortality following the mRNA (BNT162b2) and inactivated (CoronaVac) SARS-CoV-2 vaccines

Analytic plan:

To compare the incidences of various adverse events of special interest (AESI) and all-cause mortality between CoronaVac (inactivated vaccine) and BNT162b2 (mRNA-based vaccine) following the first and second doses.

Design

Retrospective cohort study among people aged 18 or above with electronic medical records who were previously received at least one dose of CoronaVac or BNT162b2 in Hong Kong from 23rd February to 9th September 2021.

Cohort selection – Inverse probability of treatment weighting (IPTW)

First, we will include all vaccinated people aged 18 or above and exclude those who received mixed doses. Second, we identify two study cohorts and define observation periods. a) First dose recipients cohort. All vaccinated people will be included. We set the date of the first dose as the start of the observation period and followed them until death, second dose vaccination, 21 days after the first dose, or censored on the data cutoff date (30th September 2021). b) Second dose recipients cohort. People who completed the vaccine series will be included in this cohort. We set the date of the second dose as the start of the observation period and follow them until death, 21 days after the first dose, or censored on the data cutoff date (30th September 2021). Third, propensity score models will be performed based on age, sex, any previous SARS-CoV-2 infection, pre-existing comorbidities, medication use, venue for vaccination, and dosing interval. Fourth, we will apply IPTW using the propensity scores and truncate the 1st and 99th percentiles of observed PS weighting distribution to account for extreme weights. Standardized mean differences of <0.1 in the variables are considered an indication of balance.

Exposure

Vaccination with CoronaVac (reference) or BNT162b2.

Outcome

Overall AESI and individual AESI are the primary outcomes, and all-cause mortality is the secondary outcome. We will use the list of AESI, which are advocated by the WHO Global Advisory Committee on Vaccine Safety. AESI outcomes include Guillain-Barré syndrome, acute disseminated encephalomyelitis, sleeping disturbance or disorder, acute aseptic arthritis, type 1 diabetes, thrombocytopenia, subacute thyroiditis, microangiopathy, heart failure, stress cardiomyopathy, arrhythmia, carditis, thromboembolism, coronary artery disease, myocardial infarction, venous thromboembolism, arterial thromboembolism, hemorrhagic disease, single organ cutaneous vasculitis, acute liver injury, acute kidney injury, acute pancreatitis, generalized convulsion, meningoencephalitis, transverse myelitis, bell's palsy, acute respiratory distress syndrome, erythema multiforme, chilblain - like lesions, anosmia and ageusia, anaphylaxis, multisystem inflammatory syndrome in children, sudden death, rhabdomyolysis. The list of AESI is defined by the International Classification of Diseases, Ninth Revision, Clinical Modification (ICD-9-CM) Diagnosis and Procedure codes, and laboratory parameters (S1 Table).

Analysis

Cohort characteristics will be tabulated. Cumulative incidence and crude incidence rate of each outcome by first and second dose will be calculated. The incidence rate ratio will be estimated to show the differences in the risks of AESI and all-cause mortality between brands of the vaccines.

IPTW-weighted Poisson regression models with person-years as an offset term will be fitted to estimate the incidence rate ratios. Sensitivity analysis will be conducted to exclude people with previous SARS-CoV-2 infections who might have an increased risk of various disease diagnoses associated with SARS-CoV-2 infection.
