# Supplementary material for: Adverse events of special interest and mortality following vaccination with mRNA (BNT162b2) and inactivated (CoronaVac) SARS-CoV-2 vaccines in Hong Kong: A retrospective study
Source: PLoS Med. 2022 Jun 21;19(6):e1004018. doi: 10.1371/journal.pmed.1004018 (PMC9212142; doi:10.1371/journal.pmed.1004018)
Supplement: Attachment — Submitted filename: PMEDICINE-D-21-05271R2 Response to reviewers 20220321.docx [file pmed.1004018.s009.docx]

21 March 2022

Dr Caitlin Moyer

Associate Editor

PLOS Medicine

**RE: PMEDICINE-D-21-05271R1**

**Manuscript Title: Adverse Events of Special Interest and mortality following the mRNA (BNT162b2) and inactivated (CoronaVac) SARS-CoV-2 vaccines: A retrospective study**

Dear Dr Moyer,

On behalf of my co-authors, I would like to thank the editors and reviewers for their valuable comments and suggestions. Responding to these, we have made the necessary adjustments in our revised manuscript, and hope that you will now find it suitable for publication. We provide our point-to-point responses to the earlier version of this paper reviewed.

**Requests from the editors:**

1. From the academic editor: Please include additional high-level contextualization of the data in the Abstract, Results, and Discussion sections. Specifically, please discuss that less than 1% of vaccine doses were associated with an AESI, with the overall AESI rate slightly higher in CoronaVac compared to BNT162b2.

*Response: Thank you for the suggestion. We have now included contextualization of our data in the Abstract (page 3 lines 71-73), Results (page 16-17 lines 353-355; page 17 lines 364-368; page 18 lines 383-389), and Discussion (page 19 lines 409-413; page 25 lines 549-551) sections, specifying the rare occurrence of overall AESI at cumulative incidence rates of 0.06-0.09% of the vaccine doses administered. We also indicate the slightly higher crude incidence rates of overall AESI among CoronaVac versus BNT162b2 recipients for both the first and second dose analyses; however, the incidence rates were comparable between the two vaccines after weighting.*

2. Title: Please revise your title according to PLOS Medicine's style. Your title must be nondeclarative and not a question. It should begin with main concept if possible. "Effect of" should be used only if causality can be inferred, i.e., for an RCT. Please place the study design ("A randomized controlled trial," "A retrospective study," "A modelling study," etc.) in the subtitle (ie, after a colon).

*Response: The title is now revised to “Adverse Events of Special Interest and mortality following the mRNA (BNT162b2) and inactivated (CoronaVac) SARS-CoV-2 vaccines: A retrospective study”.*

3. Data availability statement: Please clarify the availability of vaccination record data owned by the Department of Health in Hong Kong. If the data are owned by a third party but freely available upon request, please note this and state the owner of the data set and contact information for data requests (web or email address). Note that a study author cannot be the contact person for the data. If the data are not freely available, please describe briefly the ethical, legal, or contractual restriction that prevents you from sharing it. Please also include an appropriate contact (web or email address) for inquiries (again, this cannot be a study author).

*Response:* *The data used in this study are not freely available. Approvals for the use of data were obtained from the Department of Health and the Hospital Authority specifically for this COVID-19 vaccine safety monitoring study. We are bound by ethical, legal and contractual conditions imposed by both Department of Health and the Hospital Authority, and are not allowed to use the data for any other purposes or divulge the data to any third parties.*

*The vaccination record data are owned by the Department of Health. Clinical records are owned by Hospital Authority. Vaccination records were linked to clinical records on de-identified patients of the Hospital Authority. Following approvals from the Institutional Review Board, data requests were submitted and assessed by both Department of Health and Hospital Authority prior to data release for use by specified research delegates only. For further information regarding the data request and approval process, please see: (*[*https://www3.ha.org.hk/data/Provision/Submission*](https://www3.ha.org.hk/data/Provision/Submission)*). We have added the corresponding statements under ‘Availability of data and materials’ on page 26.*

4. Competing Interests: Several authors report research funding or grants from Pfizer, the makers of the BNT162b2 vaccine. For authors with ties to industry, please indicate whether any of the interests has a financial stake in the results of the current study.

*Response: Authors who report research funding or grants from Pfizer outside the submitted work have no financial conflict of interests to the current study. This information has been stated under ‘Competing Interests’ on pages 27-28.*

5. Abstract: Background: Please provide an additional sentence of context of why the study is important.

*Response: We have now added sentences to address the context of why the study is important is now provided in the Background of Abstract.*

*Relevant section in the Background of Abstract: “Safety monitoring of coronavirus disease 2019 (COVID-19) vaccines is crucial during massive rollout to inform the choice of vaccines and reduce vaccine hesitancy.* *Considering the scant evidence directly comparing the safety profile between mRNA and inactivated SARS-CoV-2 vaccines, this territory-wide cohort study aims to compare the incidences of various adverse events of special interest (AESI) and all-cause mortality between CoronaVac (inactivated vaccine) and BNT162b2 (mRNA-based vaccine). Our results can help vaccine recipients to make an informed choice.” (page 2 lines 31-37)*

6. Abstract: Methods and Findings: Please also present some of the specific AESI and mortality results for first and second doses of each vaccine (e.g. rates overall for AESI and mortality, and for some of the more common AESIs such as thromboembolism). Please also present results for some of the AESIs for which the two vaccines were generally comparable and more frequently reported.

*Response: Our results are now elaborated under the Methods and Findings of Abstract, including the crude incidence rates of the most commonly reported AESI after the first and second doses of CoronaVac and BNT162b2 (i.e. thromboembolism), as well as the IRRs of overall AESI and mortality comparing between the two vaccine types for both the first and second dose analyses (and any significant differences observed for specific AESI).*

*Relevant text in the Methods and Findings of Abstract: “Among 2,333,379 vaccinated individuals aged 18 or above, the first dose analysis included 1,308,820 BNT162b2 and 955,859 CoronaVac recipients, while the second dose analysis included 1,116,677 and 821,560 individuals, respectively.* *The most frequently reported AESI among CoronaVac and BNT162b2 recipients was thromboembolism (first dose: 431 vs 290 per 100,000 person-years; second dose: 385 vs 266 per 100,000 person-years). After the first dose, incidence rates of overall AESI (IRR=0.98, 95%CI=0.89–1.08) and mortality (IRR=0.96, 95%CI=0.63–1.48) associated with CoronaVac were generally comparable to BNT162b2, except for Bell’s palsy (IRR=1.95, 95%CI=1.12–3.41), anaphylaxis (IRR=0.34, 95%CI=0.14–0.79), and sleeping disturbance or disorder (IRR=0.66, 95%CI=0.49–0.89). After the second dose, incidence rates of overall AESI (IRR=0.97, 95%CI=0.87–1.08) and mortality (IRR=0.85, 95%CI=0.51–1.40) were comparable between CoronaVac and BNT162b2 recipients, with no significant differences observed for specific AESI.” (page 3 lines 53-64)*

7. Abstract: Conclusion: We suggest beginning the first sentence with: "In this study, we observed ..." and we suggest including a sentence to address the study implications.

*Response: Thank you for your suggestion. We have revised the first sentence accordingly, and added one sentence “Our results could help inform the choice of inactivated COVID-19 vaccines mainly administered in developing countries with large populations, in comparison to the safety of mRNA vaccines.”*

*The text in the Abstract Conclusion now reads as follows: “In this study, we observed that the incidences of AESI (cumulative incidence rate of 0.06-0.09%) and mortality following the first and second doses of CoronaVac and BNT162b2 vaccination were very low. Their safety profiles were generally comparable, except for a significantly higher incidence rate of Bell’s palsy, yet lower incidence rates of anaphylaxis and sleeping disturbance or disorder following first dose CoronaVac versus BNT162b2. Our results could help inform the choice of inactivated COVID-19 vaccines mainly administered in developing countries with large populations, in comparison to the safety of mRNA vaccines.* *Long-term surveillance on the safety profile of COVID-19 vaccines should continue.” (pages 3-4 lines 71-79)*

8. Author summary: At this stage, we ask that you include a short, non-technical Author Summary of your research to make findings accessible to a wide audience that includes both scientists and non-scientists. The Author Summary should immediately follow the Abstract in your revised manuscript. This text is subject to editorial change and should be distinct from the scientific abstract. Please see our author guidelines for more information: https://journals.plos.org/plosmedicine/s/revising-your-manuscript#loc-author-summary

*Response: An Author Summary is now added to the manuscript, immediately following the Abstract, as follows:*

***Why was this study done?***

- *Various SARS-CoV-2 vaccines have been developed for coronavirus disease 2019 (COVID-19) prevention to reduce the risks of severe disease and death; however, vaccine hesitancy due to fear of adverse reactions remains a barrier to mass uptake.*
- *Continuous post-marketing surveillance of vaccine safety is crucial to guide informed decision-making and promote vaccine uptake in the community.*
- *There is very limited evidence directly comparing the safety profile between mRNA-based and inactivated SARS-CoV-2 vaccines.*

***What did the researchers do and find?***

- *A territory-wide, retrospective cohort study was conducted on individuals who had received at least one dose of BNT162b2 (mRNA-based vaccine, Comirnaty) or CoronaVac (inactivated SARS-CoV-2 vaccine) from 23^rd^ February to 9^th^ September 2021 in Hong Kong, to compare the occurrence of selected adverse events of special interest (AESI) and all-cause death between recipients of the two vaccines during 21 days of follow-up after the first and second doses, respectively.*
- *The incidence of overall AESI was very low for both vaccines (cumulative incidence rate of 0.06-0.09%); and the most frequently reported AESI among CoronaVac and BNT162b2 recipients was thromboembolism (first dose: 431 vs 290 per 100,000 person-years; second dose: 385 vs 266 per 100,000 person-years).*
- *The incidence rates of overall AESI and all-cause death were comparable between CoronaVac and BNT162b2 recipients after the first and second doses; except for a higher incidence rate of Bell’s palsy, and lower incidence rates of anaphylaxis and sleeping disturbance or disorder following first dose CoronaVac versus BNT162b2.*

***What do these findings mean?***

- *Both vaccines had similarly acceptable safety profiles, and the risks of AESI and all-cause death following CoronaVac or BNT162b2 vaccination were very low, which would add real-world evidence to the safety of both COVID-19 vaccines, and help reduce vaccine hesitancy by addressing the safety concerns.*
- *Our results would inform the public about the choice of COVID-19 vaccines, and governments about the allocation of healthcare resources for monitoring specific AESI during vaccine rollout (especially for Bell’s palsy among first dose CoronaVac recipients, and anaphylaxis and sleeping disturbance or disorder among first dose BNT162b2 recipients).*
- *As currently done for mRNA vaccines, clinical data and observational reports of all COVID-19 vaccines should be made publicly available in a timely manner, and long-term monitoring of their safety profile should continue.*

*This has been inserted on pages 5-6, lines 83-118.*

9. Throughout: In-text citations: Please place in-text citations within square brackets, placed before the sentence punctuation, for example [1]. Where multiple references are cited, please do not include spaces within brackets.

*Response: The above in-text citation formatting has been applied to the revised manuscript text throughout.*

10. Methods: Please ensure that the study is reported according to the STROBE guideline. Please add the following statement, or similar, to the Methods: "This study is reported as per the Strengthening the Reporting of Observational Studies in Epidemiology (STROBE) guideline (S1 Checklist)." The STROBE guideline can be found here: http://www.equator-network.org/reporting-guidelines/strobe/

*Response: The above statement on reporting as per the STROBE guideline (S1 Checklist) has been added to the Methods section (page 16 lines 331-332).*

11. Methods: Did your study have a prospective protocol or analysis plan? Please state this (either way) early in the Methods section.

a) If a prospective analysis plan (from your funding proposal, IRB or other ethics committee submission, study protocol, or other planning document written before analyzing the data) was used in designing the study, please include the relevant prospectively written document with your revised manuscript as a Supporting Information file to be published alongside your study, and cite it in the Methods section. A legend for this file should be included at the end of your manuscript.

*Response: Thank you for your suggestion. This is a regulatory authority-initiated (Department of Health) pharmacovigilance programme due to emergency use of COVID-19 vaccine. The overall aim of this programme is to evaluate safety profiles of the COVID-19 vaccines in Hong Kong; hence each study is designed in response to the local need. Consequently, a prospective study protocol or analysis plan was not separately developed in the design of this study. However, this study was part of an overall research programme which was assessed by the regulatory authority (Department of Health), clinical data custodian (Hospital Authority), Institutional Review Board, and funding body (Food and Health Bureau). We developed a protocol when designing this study, which was followed and used for independent double checking of data analysis to ensure data quality and reproducibility. We have now included the protocol with the revised manuscript as Supporting Information (S1 Protocol), and cited the protocol in the Methods section (page 14 lines 291-298).*

b) If no such document exists, please make sure that the Methods section transparently describes when analyses were planned, and when/why any data-driven changes to analyses took place.

*Response: We have uploaded the protocol document as Supporting Information (S1 Protocol), and* *added the corresponding statements about analysis plan in the Methods section (page 14 lines 291-300).*

c) In either case, changes in the analysis-- including those made in response to peer review comments-- should be identified as such in the Methods section of the paper, with rationale.

*Response:* *We have* *added details of the changes made in response to peer review comments, i.e. assessments of model goodness of fit included of the Poisson regression models, in the Methods section, as follows: “Goodness-of-fit of Poisson regression models were assessed by deviance chi-square test and overdispersion test, which were made during the revision in according to referee’s comments [1].” (page 15 lines 323-325)*

Reference:

1. Fávero LPL, Belfiore P, Santos MAd, Souza RF. Overdisp: A Stata (and Mata) Package for Direct Detection of Overdispersion in Poisson and Negative Binomial Regression Models. Statistics, Optimization & Information Computing. 2020;8(3):773-89.

12. Methods: Line 131: Please clarify as (Figure 1) here.

*Response: The figure is now renamed as Fig 1 in the revised manuscript.*

13. Methods: Line 172-175: Please explain here exactly how the AESI outcomes were adapted from the WHO Global Advisory Committee on Vaccine Safety list of AESI (e.g. the specific rationale for adding to the conditions listed in Table 4 of reference 28).

*Response: AESI outcomes were adapted from the WHO Global Advisory Committee on Vaccine Safety and used by the EMA-funded ACCESS (vACCine covid-19 monitoring readinESS) project [1,2].* *The list was initially created by the CEPI-funded SPEAC (Safety Platform for Emergency vACcines) project as part of the harmonization of COVID-19 vaccine safety monitoring during the clinical development phase of the vaccines [3]. The SPEAC project updated the list on 23^rd^ December 2020 to add three more AESI outcomes based on the latest literature: 1)* *subacute thyroiditis, 2) pancreatitis, and 3) rhabdomyolysis.* *These three AESI outcomes have not yet been endorsed by the WHO but were included in this study following discussion and consensus reached between co-investigators and the regulatory authority. All AESI outcomes have been identified as potentially related to existing marketed vaccines, events related to vaccine platforms or adjuvants, and events that may be associated with COVID-19.* *We have elaborated the above rationale for including the current list of events in AESI outcomes in the Methods section.*

*Relevant text in the Outcomes of Methods section: “Our study outcomes adapted the full list of AESI outcomes endorsed by the WHO Global Advisory Committee on Vaccine Safety, which has been widely used for the safety surveillance of COVID-19 vaccines; a range of events suggested by the EMA-funded vACCine covid-19 monitoring readinESS (ACCESS) project; and ‘subacute thyroiditis’, ‘pancreatitis’, and ‘rhabdomyolysis’ updated by the CEPI-funded Safety Platform for Emergency vACcines (SPEAC) project [1-3].* *The latter three AESI outcomes have not yet been endorsed by the WHO but were included in this study following discussion and consensus reached between co-investigators and the regulatory authority. All AESI outcomes have been identified as potentially related to existing marketed vaccines, events related to vaccine platforms or adjuvants, and events that may be associated with COVID-19.” (page 12 lines 238-247)*

References:

1. World Health Organization. COVID-19 Vaccines: Safety Surveillance Manual - Module: Establishing active surveillance systems for adverse events of special interest during COVID-19 vaccine introduction 2021 [30/9/2021]. Available from: <https://www.who.int/vaccine_safety/committee/Module_AESI.pdf>.

2. vACCine covid-19 monitoring readinESS (ACCESS). Protocol – Background rates of Adverse Events of Special Interest for monitoring COVID-19 vaccines Version 1.1 September 21 2020 2020 [18/3/2022]. Available from: <https://vac4eu.org/wp-content/uploads/2020/09/ACCESS_BGRprotocolSept212020.pdf>.

3. Safety Platform for Emergency vACcines (SPEAC). SO2-D2.1.2 Priority List of COVID-19 Adverse events of special interest: Quarterly update December 2020 2020 [18/3/2022]. Available from: <https://brightoncollaboration.us/wp-content/uploads/2020/12/SO2_D2.1.2_V1.2_COVID-19_AESI-update-23Dec2020.pdf>.

14. Methods: Line 185: Previous SARS-CoV-2 infection: Please provide some information as to how this covariate was assessed. In Table 1, this variable is indicated by “COVID-19 survivor” and it may help to provide a clear description of the definition of this measure.

*Response: Thank you for the question.* *Previous SARS-CoV-2 infection was defined as “ever a positive result on the SARS‑CoV‑2 reverse transcription-polymerase chain reaction (RT-PCR) test before COVID-19 vaccination” [1]. We have now added this definition at the first mention in the Covariates subsection under Methods (page 13 lines 266-268).*

*In Table 1, we have changed the term “COVID-19 survivor” to “Previous SARS-CoV-2 infection” for consistency.*

Reference:

1. Lai FTT, Huang L, Peng K, Li X, Chui CSL, Wan EYF, et al. Post-Covid-19-vaccination adverse events and healthcare utilization among individuals with or without previous SARS-CoV-2 infection. J Intern Med. 2022.

15. Methods: Line 197: Please provide more specific information on how covariates “were taken into account” here.

*Response: Thank you for the question. We have now clarified that the covariates of vaccine recipients were “weighted between BNT162b2 and CoronaVac for each dose”.*

*Relevant section in text: “Age, sex, any previous SARS-CoV-2 infection (defined as ever a positive result on the SARS‑CoV‑2 reverse transcription-polymerase chain reaction [RT-PCR] test before COVID-19 vaccination), pre-existing comorbidities documented from 2018 (myocardial infarction (MI), peripheral vascular disease, cerebrovascular disease, coronary artery disease, chronic obstructive pulmonary disease, dementia, paralysis, diabetes with and without chronic complications, hypertension, chronic renal failure, mild and moderate-severe liver diseases, ulcers, rheumatoid arthritis and other inflammatory polyarthropathies, acquired immune deficiency syndrome, malignancy, and metastatic solid tumor), medication use in the past 90 days (renin-angiotensin-system agents, beta blockers, calcium channel blockers, diuretics, lipid-lowering agents, insulins, antidiabetic drugs, anticoagulants, antiplatelets, hormonal agents, antidepressants, non-steroidal anti-inflammatory drugs, drugs for gout, antiepileptic drugs, antiviral drugs, antibacterial drugs, and immunosuppressants), venue for vaccination (community vaccination centers, clinics, and others), and the interval of administration between the first and second doses of vaccine recipients were weighted between BNT162b2 and CoronaVac for each dose (as elaborated in the Statistical analyses below).” (page 13 lines 266-281)*

16. Methods: Line 203: Please clarify how “history of AESI” is defined (for example, does this indicate a history of any of the conditions listed in Table S1, over the same time period over which other covariates/comorbidities were considered?).

*Response: “History of AESI” was defined as the history of any of the conditions, except mortality, listed in S1 Table from 2005 to the date of first dose COVID-19 vaccination. This is now elaborated in the Methods section.*

*Relevant section in text: “History of AESI was defined as the history of any of the conditions, except mortality, listed in S1 Table from 2005 to the date of first dose COVID-19 vaccination.” (page 14 lines 304-306)*

17. Results: Line 272: “Nevertheless, none of the AESI investigated had demonstrated significant differences in the incidence rate comparing between second dose CoronaVac and BNT162b2 (Table 4).” It may be useful here to point out the results for comparisons for Bell’s palsy, anaphylaxis, and sleeping disturbance/disorder (those AESIs that differed significantly for the first dose).

*Response: Thank you for the suggestion. This is now elaborated in the Results.*

*Relevant section in text: “In contrast to the significant differences observed for Bell’s palsy (higher incidence rate for CoronaVac), anaphylaxis and sleeping disturbance or disorder (higher incidence rates for BNT162b2) following the first dose, none of the AESI investigated had demonstrated significant differences in the incidence rate comparing between second dose CoronaVac and BNT162b2 (Table 3).” (page 18 lines 395-399)*

18. Discussion: Please present and organize the Discussion as follows: a short, clear summary of the article's findings; what the study adds to existing research and where and why the results may differ from previous research; strengths and limitations of the study; implications and next steps for research, clinical practice, and/or public policy; one-paragraph conclusion.

*Response: The Discussion section is now presented and re-organized as follows: a short, clear summary of the article's findings (paragraph 1, page 19); what the study adds to existing research and where and why the results may differ from previous research (paragraphs 2-6, pages 19-23); strengths and limitations of the study (paragraph 7, pages 23-24); implications and next steps for research, clinical practice, and/or public policy (paragraph 8, page 24); and one-paragraph conclusion (paragraph 9, page 25). The heading of ‘Conclusion’ is now removed as the one-paragraph conclusion is included under the Discussion section (page 25 lines 549-557).*

19. References: Please use the "Vancouver" style for reference formatting, and please check each reference for NLM abbreviations for journal titles. Please see our website for other reference guidelines https://journals.plos.org/plosmedicine/s/submission-guidelines#loc-references

*Response: The Vancouver reference style has been adopted in the revised manuscript.*

20. Table 1: Please provide numbers in addition to percentages. Please clarify if “Overall AESI” under “Pre-existing comorbidities” refers to the history of AESI conditions, prior to vaccination.

*Response: Thank you for the suggestion. Numbers are now provided alongside the percentages in Table 1. “Overall AESI” refers to the history of AESI prior to COVID-19 vaccination, except mortality. We have now changed “Overall AESI” to “History of AESI” for consistency.*

21. Tables 2 and 3: Please describe in the legend, the rationale for unreported incidence (for example, for ADEM).

*Response: Thank you for the clarification. Cumulative incidence and crude incidence rate of some AESI were not reported when the number of events for that AESI was zero. This has been added to the footnote of Tables 2 and 4 (previous Table 3 is now renumbered as Table 4, as cited in ascending numerical order in the main text).*

22. STROBE Checklist: Thank you for including the STROBE Checklist. Please revise the checklist, referring to locations within the text by section and paragraph numbers, rather than page numbers (for example, Methods, Paragraph 1).

*Response: Thank you for the reminder. The STROBE Checklist (S1 Checklist) is now revised.*

**Comments from the reviewers:**

**Reviewer #1**: This is an extremely original article, given the fact that comparison of the coronavac and BNT162b2 Covid vaccine safety can only be done in a very few countries, among which Hong Kong has the lead. I have the following comments:

*	Introduction. Among the different tools to compare Covid vaccines' safety, disproportionality analysis on the WHO pharmacovigilance database is a non specific way to generate safety signals. Please introduce the fact that this cannot be done to date for the Coronavac given that there is an incomplete and selective pharmacovigilance signals reporting Coronavac, which is not the case for the mRNA vaccines (Lancet Infect Dis. 2021 Nov; 21(11): 1490-1491. doi: 10.1016/S1473-3099(21)00646-0.).

*Response: Thank you very much for the suggestion. We have now provided such background in the Introduction section.*

*Relevant text in Introduction: “This was in line with a disproportionality analysis that used the World Health Organization (WHO) pharmacovigilance database and concluded that a signal of facial paralysis had not been displayed for mRNA COVID-19 vaccines when compared to other viral vaccines [1]. While the WHO pharmacovigilance database may be used for generating safety signals for further testing and confirmation, it relies on the spontaneous reporting of suspected adverse drug reactions by patients and health professionals of participating countries, which has been extensively performed for BNT162b2 but not as much for CoronaVac [2].” (pages 8-9 lines 166-173)*

References:

1. Renoud L, Khouri C, Revol B, Lepelley M, Perez J, Roustit M, et al. Association of Facial Paralysis With mRNA COVID-19 Vaccines: A Disproportionality Analysis Using the World Health Organization Pharmacovigilance Database. JAMA Internal Medicine. 2021;181(9):1243-5.

2. Khouri C, Roustit M, Cracowski J-L. Adverse event reporting and Bell's palsy risk after COVID-19 vaccination. The Lancet Infectious Diseases. 2021;21(11):1490-1.

*	Page 5, line 99 to 110 needs complete rewriting. Here, there is an heterogenous mixture of signal detection and signal confirmation. We have very strong pharmacoepidemiological data for BNT162b2 Covid supporting lymphadenopathy, myocarditis, pericarditis, herpes zoster infections, and potentially appendicitis, but not the other (<http://www.nejm.org/doi/10.1056/NEJMoa2110475>).

*Response: Thank you for the suggestion. The corresponding sentences are now revised.*

*Relevant text in Introduction: “Besides, strong pharmacoepidemiological data have suggested an excess risk of myocarditis and pericarditis [1-3], lymphadenopathy, herpes zoster infection, and potentially appendicitis with this mRNA vaccine [1].” (page 8 lines 153-156)*

*Furthermore, our previous studies identified significantly increased risk of acute myocarditis or pericarditis among male adolescents following BNT162b2 vaccination, notably after the second dose. We have now elaborated on the risks of myocarditis and pericarditis among adolescents in Hong Kong following BNT162b2 vaccination under Discussion.*

*Relevant text in Discussion: “The small number of carditis cases in this study may be attributed to our exclusion of vaccinated individuals under the age of 18 years old, as a significantly increased risk of acute myocarditis or pericarditis has been identified among male adolescents following BNT162b2 vaccination in local studies, notably after the second dose [4-6].” (page 22 lines 480-484)*

References:

1. Barda N, Dagan N, Ben-Shlomo Y, Kepten E, Waxman J, Ohana R, et al. Safety of the BNT162b2 mRNA Covid-19 Vaccine in a Nationwide Setting. New England Journal of Medicine. 2021;385(12):1078-90.

2. Klein NP, Lewis N, Goddard K, Fireman B, Zerbo O, Hanson KE, et al. Surveillance for Adverse Events After COVID-19 mRNA Vaccination. JAMA. 2021;326(14):1390-9.

3. Montgomery J, Ryan M, Engler R, Hoffman D, McClenathan B, Collins L, et al. Myocarditis Following Immunization With mRNA COVID-19 Vaccines in Members of the US Military. JAMA Cardiology. 2021;6(10):1202-6.

4. Lai FTT, Li X, Peng K, Huang L, Ip P, Tong X, et al. Carditis After COVID-19 Vaccination With a Messenger RNA Vaccine and an Inactivated Virus Vaccine. Annals of Internal Medicine. 2022;175:362-70. doi: 10.7326/M21-3700.

5. Li X, Lai FTT, Chua GT, Kwan MYW, Lau YL, Ip P, et al. Myocarditis Following COVID-19 BNT162b2 Vaccination Among Adolescents in Hong Kong. JAMA Pediatrics. 2022. doi:10.1001/jamapediatrics.2022.0101.

6. Chua GT, Kwan MYW, Chui CSL, Smith RD, Cheung EC-L, Ma T, et al. Epidemiology of Acute Myocarditis/Pericarditis in Hong Kong Adolescents Following Comirnaty Vaccination. Clinical Infectious Diseases. 2021:ciab989. doi: 10.1093/cid/ciab989.

*	Page 6, line 128. Please specify the source of BNT162b2 vaccines, are those tozinameran?

*Response: Thank you for the question. We have now elaborated on the supply of vaccines in Hong Kong on page 9 lines 185-188.*

*Relevant text in Methods: “A territory-wide cohort study was conducted to compare the incidence rates of AESI and all-cause mortality between CoronaVac (supplied by Sinovac) and BNT162b2 (supplied by Fosun Pharma as Comirnaty vaccines, manufactured by BioNTech in Germany) [1] vaccination after the first and second doses, respectively.”*

*According to the vaccine information provided by the Hong Kong government, both Fosun-BioNTech and Pfizer-BioNTech produce mRNA vaccines using the technology of BioNTech [1]; hence,* *Fosun-BioNTech Comirnaty available in Hong Kong is equivalent to Pfizer-BioNTech Comirnaty (tozinameran).*

Reference:

1. The Government of the Hong Kong Special Administrative Region. Early Vaccination for All - About the Vaccines: The Government of the Hong Kong Special Administrative Region,; 2022 [updated 2/3/2022. Available from: <https://www.covidvaccine.gov.hk/en/vaccine>.

*	The nature of the sleeping disturbances following the first BNT162b2 dose is intriguing and not previously reported, could you check whether this is correlated to the initial reactogenicity?

*Response: Thank you for your question. Despite the slightly higher incidence rate of sleeping disturbance or disorder among first dose BNT162b2 versus CoronaVac recipients (167 vs 131 per 100,000 person-years), the distribution of occurrence of sleeping disturbance or disorder within 21 days after the first dose of BNT162b2 is illustrated below (and S3 Fig). Days of onset of sleep disturbance or disorder were not intensive shortly after vaccination. Hence its occurrence might not be correlated to initial reactogenicity. The corresponding sentences are now added in the Results section.*

*Relevant text in Results: “Despite the slightly higher incidence rate of sleeping disturbance or disorder among first dose BNT162b2 versus CoronaVac recipients, days of onset of this AESI were not intensive shortly after BNT162b2 vaccination (S3 Fig). Hence its occurrence might not be correlated to initial reactogenicity.” (page 17 lines 371-374)*

*As we could not determine the onset or duration of sleeping disturbances following BNT162b2 vaccination from the electronic medical records in the database, it is also possible that individuals might not have sought medical attention promptly for such condition. Based on our best knowledge and the current literature, there is a lack of evidence supporting the correlation between sleeping disturbances and initial reactogenicity following BNT162b2 vaccination. We agree that further investigation into any biological or immunological mechanisms about sleeping disturbances following COVID-19 vaccination is needed. The corresponding sentences are now added to the Discussion section.*

*Relevant text in Discussion:* “*In terms of the higher incidence rate of sleeping disturbance or disorder following the first BNT162b2 dose, there is a lack of evidence suggesting a correlation with initial reactogenicity. Based on our best knowledge and the current literature, we are unable to identify any biological or immunological mechanisms to explain sleeping disturbances following COVID-19 vaccination, hence further investigation is needed.” (page 21 lines 468-473)*

*	One major interest of the study is the population-based cohort, and the ability to try to take into account a larger variety of confounding bias. However, residual biases are still likely given that the initial population strikingly differs. The authors acknowledged that in the discussion. I do not need to see E-values here

*Response: Thank you for the acknowledgement. We have already addressed the potential bias of residual confounding as our first study limitation.*

*Relevant text in Discussion:* “*First, similar to all epidemiological studies, the demographic and clinical characteristics of vaccine recipients were balanced with PS weighting at baseline, residual confounding could still exist.” (page 23 lines 521-523)*

*	Overall, the study adds few to the numerous data already available for BNT162b2 Covid vaccine safety. The major interest is the comparison with Coronavac, for which data are new. I think that the discussion could briefly introduce the fact that publicly available reports of continuous monitoring of vaccine's safety through pharmacovigilance and pharmacoepidemiology, as done for mRNA vaccines is mandatory for all vaccines platforms.

*Response: Thank you for the suggestion. We have now emphasized this in paragraph 8 of the Discussion section.*

*Relevant text in Discussion: “By evaluating and comparing the safety profile of CoronaVac against BNT162b2 (Comirnaty), the first FDA approved SARS-CoV-2 vaccine for COVID-19 prevention, this study could help inform the choice of inactivated COVID-19 vaccines mainly administered in developing countries with large populations, in comparison to the safety of mRNA vaccines. Despite the slightly higher crude incidence rates of overall AESI among CoronaVac versus BNT162b2 recipients, the two vaccine platforms had a similar safety profile after adjusting for baseline characteristics. As currently done for mRNA vaccines, clinical data and observational reports of COVID-19 vaccines should continue to be made publicly available for all vaccine platforms in a timely manner [1,2], as a means of facilitating the continuous monitoring of vaccine safety via pharmacovigilance and pharmacoepidemiology.” (page 24 lines 535-544)*

References:

1. LKS Faculty of Medicine Department of Pharmacology & Pharmacy. COVID-19 Vaccines Adverse Events Response and Evaluation (CARE) Programme 2022 [18/3/2022]. Available from: <https://www.hkcare.hku.hk/>.

2. LKS Faculty of Medicine Department of Pharmacology & Pharmacy. COVID-19Hub - Our contribution to the prevention and treatment of COVID-19 2022 [18/3/2022]. Available from: <https://www.pharma.hku.hk/en/COVID19Hub/COVID-19-Main>.

**Reviewer #2**: Thanks for the opportunity to review your manuscript. My role is as a statistical reviewer so my comments focus on the study design, data and analysis presented in the manuscript. I have put general comments first, and followed these with queries relevant to a specific section of the manuscript (with a page/line reference).

This is a retrospective cohort study based in the Hong Kong SAR in China, comparing AESI and mortality between people who received the Pfizer-BioNTech to those that received the CoronoVac/Sinovac Covid-19 vaccine. The temporal window for risk of the outcomes was examined for several definitions - the main being from data of either first or second vaccination. Propensity scores were estimated and then used to weight the cohort to balance on the observed covariates. The outcomes were a list of particular vaccine safety AESIs, and all-cause mortality. Poisson regression was used to estimate IRRs for the AESIs and mortality. The study included data from Feb 2021 to- September 2021 - the data is very fresh. The IRR showed similar overall rates of the selected AESIs between vaccines (and can exclude any large or medium differences) with some differences for particular AESIs. In theory mortality is a competing outcome for the AESIs, but mortality was rare enough over the study period it would be highly unlikely to bias the results. The sensitivity analysis showed broadly similar results - I note that AKIs in in the sensitivity analysis showed a difference between groups but these were uncommon and the IRR CI interval is quite wide.

*Response: Thank you very much for reviewing this manuscript thoroughly, and the accurate summary of our study findings. Regarding the results of acute kidney injury (AKI) in the sensitivity analysis of excluding people with previous SARS-CoV-2 infection (S3 Table), they were in line with the main analysis with no significant differences between CoronaVac and BNT162b2 groups after the first or second dose. The wider confidence intervals in the results of the sensitivity analysis may be due to the small number of AKI events following COVID-19 vaccination. The corresponding sentences are now added to the Discussion section.*

*Relevant text in Discussion: “Besides, the wider confidence intervals in the results of the sensitivity analysis of acute kidney injury suggested the difference was not significant, possibly caused by small number of events following COVID-19 vaccination.” (page 19 lines 418-420)*

One thing I would like clarified - there introduction states the choice of vaccine was up to the individual, but where there any guidelines that some people should get a particular vaccine or differences in service delivery that might lead to a difference in availability?

*Response: Thank you for your question. Certain patient groups were prioritized to receive vaccination at the start and specific times of the community vaccination programme. However, there were no guidelines that recommend certain individuals or patient groups should receive particular vaccine or differences in service delivery. Individuals remain free to choose their preferred type of vaccine at their first dose, yet they are unable to switch vaccine type for their second dose (except for rare instances of anaphylaxis following the first dose, where switching between vaccine types may be allowed on a case-by-case basis) [1]. In our study, individuals who had received BNT162b2 as the first dose followed by CoronaVac as the second dose (or vice versa) were excluded in the second dose analysis. These are now elaborated in the Methods section.*

*Relevant text in Methods: “The mass COVID-19 vaccination programme in Hong Kong was launched on 23^rd^ February 2021 for CoronaVac and 6^th^ March 2021 for BNT162b2. Certain patient groups were prioritized to receive vaccination at the start and specific times of the community vaccination programme. However, there were no guidelines that recommend certain individuals or patient groups should receive particular vaccine or differences in service delivery. Individuals remain free to choose their preferred type of vaccine at their first dose, yet they are unable to switch vaccine type for their second dose (except for rare instances of anaphylaxis following the first dose, where switching between vaccine types may be allowed on a case-by-case basis).” (page 10 lines 190-197)*

Reference:

1. The Government of the Hong Kong Special Administrative Region. Early Vaccination for All - FAQs: The Government of the Hong Kong Special Administrative Region,; 2022 [updated 2/3/2022. Available from: <https://www.covidvaccine.gov.hk/en/faq>.

Was a protocol or statistical analysis plan developed for the study? If so, can this be made available as part of the review?

*Response: Thank you for the question. This is a regulatory authority-initiated (Department of Health) pharmacovigilance programme due to emergency use of COVID-19 vaccines. The overall aim of this programme is to evaluate the safety profile of the COVID-19 vaccines in Hong Kong; hence each study is designed in response to the local need. Consequently, a prospective study protocol or analysis plan was not separately developed in the design of this study. However, this study was part of an overall research programme which was assessed by the regulatory authority (Department of Health), clinical data custodian (Hospital Authority), Institutional Review Board, and funding body (Food and Health Bureau). We developed a protocol (S1 Protocol) when designing this study, which was followed and used for independent double checking of data analysis to ensure data quality and reproducibility. We have added the corresponding statements about analysis plan in the Methods section (page 14 lines 291-298).*

P6, L137. Was the unique mapping key a health insurance ID or similar?

*Response: The Hong Kong Identity Card number or foreign passport number was the unique mapping key. This is now elaborated in the Methods section.*

*Relevant text in Methods: “Anonymized, population-wide COVID-19 vaccination records obtained from the Department of Health and electronic medical records retrieved from the Hong Kong Hospital Authority (the statutory body managing public healthcare services in the region) were linked using a unique mapping key (Hong Kong Identity Card number or foreign passport number).” (page 10 lines 204-207)*

P8, L173. Were the adaptions made to exclude conditions unlikely to be recorded from the EHR data available?

*Response: Thank you for the question. We did not exclude any conditions in the AESI outcomes endorsed by the WHO Global Advisory Committee on Vaccine Safety, or conditions unlikely to be recorded from the electronic health records. To avoid confusion, we have removed the phrase “with adaptations to our dataset” in the corresponding sentence under the Outcomes subsection of Methods (page 12 line 249).*

P9, L201. What criteria was used to decide which of the covariates should be included in the PS model?

*Response:* *Baseline covariates that were potential risk factors of AESI events were chosen. Age, sex, pre-existing comorbidities, and history of medication use were potential risk factors of adverse events following COVID-19 vaccination, as reported by previous studies [1-5].* *The background rate of AESI would influence both the vaccination rate and risk of AESI, hence it was added to the PS model as well. We have added the corresponding sentences in the Methods section.*

*Relevant text in Methods:* *“As reported by previous studies [1-5], baseline covariates that were potential risk factors of AESI events were chosen. Age, sex, any previous SARS-CoV-2 infection (defined as ever a positive result on the SARS‑CoV‑2 reverse transcription-polymerase chain reaction [RT-PCR] test before COVID-19 vaccination), pre-existing comorbidities documented from 2018 (myocardial infarction (MI), peripheral vascular disease, cerebrovascular disease, coronary artery disease, chronic obstructive pulmonary disease, dementia, paralysis, diabetes with and without chronic complications, hypertension, chronic renal failure, mild and moderate-severe liver diseases, ulcers, rheumatoid arthritis and other inflammatory polyarthropathies, acquired immune deficiency syndrome, malignancy, and metastatic solid tumor), medication use in the past 90 days (renin-angiotensin-system agents, beta blockers, calcium channel blockers, diuretics, lipid-lowering agents, insulins, antidiabetic drugs, anticoagulants, antiplatelets, hormonal agents, antidepressants, non-steroidal anti-inflammatory drugs, drugs for gout, antiepileptic drugs, antiviral drugs, antibacterial drugs, and immunosuppressants), venue for vaccination (community vaccination centers, clinics, and others), and the interval of administration between the first and second doses of vaccine recipients were weighted between BNT162b2 and CoronaVac for each dose (as elaborated in the Statistical analyses below).” (page 13 lines 265-281)*

References:

1. Lai FTT, Huang L, Chui CSL, Wan EYF, Li X, Wong CKH, et al. Multimorbidity and adverse events of special interest associated with Covid-19 vaccines in Hong Kong. Nature Communications. 2022;13(1):411.

2. Xiong X, Wong CKH, Au ICH, Lai FTT, Li X, Wan EYF, et al. Safety of inactivated and mRNA COVID-19 vaccination among patients treated for hypothyroidism: a population-based cohort study. Thyroid. 2022.

3. Antonelli M, Penfold RS, Merino J, Sudre CH, Molteni E, Berry S, et al. Risk factors and disease profile of post-vaccination SARS-CoV-2 infection in UK users of the COVID Symptom Study app: a prospective, community-based, nested, case-control study. Lancet Infect Dis. 2022;22(1):43-55.

4. Lai FTT, Huang L, Peng K, Li X, Chui CSL, Wan EYF, et al. Post-Covid-19-vaccination adverse events and healthcare utilization among individuals with or without previous SARS-CoV-2 infection. J Intern Med. 2022.

5. Wong CKH, Xiong X, Lau KTK, Chui CSL, Lai FTT, Li X, et al. Impact of a delayed second dose of mRNA vaccine (BNT162b2) and inactivated SARS-CoV-2 vaccine (CoronaVac) on risks of all-cause mortality, emergency department visit, and unscheduled hospitalization. BMC Medicine. 2022;20(1):119.

P9, L205. What were the observed weights of these 1/99 percentiles of the weighting distribution?

*Response:* *Thank you for your question. We have now appended the observed propensity score weighting distribution after truncation of <1st and >99th percentiles among CoronaVac and BNT162b2 recipients after each vaccine dose to S1 Fig (page 15 lines 309-311).*

P9, L208. Was common-support (sufficient overlap in PS scores) between the two groups apparent? Could a visualization of this be included in the supplementary materials please?

*Response:* *Thank you for your suggestion. The distribution of PS scores in the first and second dose of CoronaVac and BNT162b2 highly overlapped after weighting. We have now illustrated this in S2 Fig, and mentioned under the Results section.*

*Relevant text in Results: “The distribution of PS scores in the two vaccine groups after each dose highly overlapped after weighting (S2 Fig).” (page 16 lines 342-344)*

P9, L210. Were any assessments of model goodness of fit included of the Poisson regression models?

*Response:* *Thank you for your suggestion. We assessed goodness-of-fit of Poisson regression models by deviance chi-square test and overdispersion test [1]. The results demonstrated adequate model fit for all Poisson regression models. We have now illustrated this in S2 Table, and elaborated under the Methods and Results sections, respectively.*

*Relevant text in Methods: “Goodness-of-fit of Poisson regression models were assessed by deviance chi-square test and overdispersion test, which were made during the revision in according to referee’s comments [1].” (page 15 lines 323-325)*

*Relevant text in Results: “Goodness-of-fit test demonstrated adequate model fit for all Poisson regression models (S2 Table).” (page 17 lines 363-364)*

Reference:

1. Fávero LPL, Belfiore P, Santos MAd, Souza RF. Overdisp: A Stata (and Mata) Package for Direct Detection of Overdispersion in Poisson and Negative Binomial Regression Models. Statistics, Optimization & Information Computing. 2020;8(3):773-89.

Supp Appendix, Table 2 (and throughout the main tables). Even with a population-based study, 2 decimal places for the IRRs and 95% CI should be ok.

*Response: Thank you for your suggestion. We have now corrected to 2 decimal places for the IRRs and 95% CI in the manuscript, main tables, and S3 Table (previously as S2 Table).*

**Reviewer #3**: This is an impressive paper worthy of publication.

I was struck by the statement that there were no missing data in the covariates (line 199).

*Response: Thank you for the opportunity to clarify. There were no missing values in covariates, under the assumption that the absence of a medication record would imply the specific medication had neither been prescribed using electronic prescribing system nor dispensed by the Hospital Authority. Similar to other database studies, the absence of a disease diagnosis in an individual’s life-time records from the Hospital Authority database was treated as an individual without a specific disease. We have now added the corresponding sentences for elaboration in the Methods section (pages 13-14 lines 283-288).*

In the Methods it was stated that the ICD 9th Revision (ICD-9-CM) was used for definitions of AESI (line 176). Is this a more flexible definition of anaphylaxis than the Brighton criteria?

You may wish to consider a statement in the Methods or Discussion that clarifies this.

*Response: Thank you for the question. We agree with the reviewer that the definition of anaphylaxis based on the Brighton criteria is more stringent, which has two requirements for reaching levels 1, 2 & 3 of certainty: 1) there must be a ‘sudden onset’ and ‘rapid progression’ of the signs and symptoms, and 2) the signs and symptoms should involve at least two body systems [1]. Our electronic medical records did now allow access to the free text, or contact the doctors or patients to confirm the Brighton case definition of anaphylaxis and level of certainty. We have now added such study limitation to the Discussion section.*

*Relevant text in Discussion: “Lastly, our electronic medical records did not allow access to the free text, or contacts with doctors or patients; therefore, we were unable to assess these cases using the Brighton case definition of anaphylaxis and level of certainty [1].” (page 24 lines 530-533)*

Reference:

1. Safety Platform for Emergency vACcines (SPEAC). SO2- D2.5.2.1 – AESI Case Definition Companion Guide for 1st Tier AESI – Anaphylaxis: Safety Platform for Emergency vACcines (SPEAC),; 2021 [updated 5/2/2021. Available from: <https://brightoncollaboration.us/wp-content/uploads/2021/03/SPEAC_D2.5.2.1_Anaphylaxis-Case-Definition-Companion-Guide_V1.0-12070-1.pdf>.

We sincerely hope that you will consider this revised manuscript favorably. Should there be further corrections or clarifications needed, we are more than happy to make the necessary changes and provide additional information. We look forward to hearing from you soon.

Yours sincerely,

Ian Chi Kei Wong, PhD

Centre for Safe Medication Practice and Research, Department of Pharmacology and Pharmacy, LKS Faculty of Medicine, The University of Hong Kong

2/F, 21 Sassoon Road, Li Ka Shing Faculty of Medicine, Laboratory Block, Faculty of Medicine Building, Hong Kong SAR, China

Phone: (+852) 3917 9024

Fax: (+852) 2817 0859

Email: [wongick@hku.hk](mailto:wongick@hku.hk)
