# Supplementary material for: Adverse events of special interest and mortality following vaccination with mRNA (BNT162b2) and inactivated (CoronaVac) SARS-CoV-2 vaccines in Hong Kong: A retrospective study
Source: PLoS Med. 2022 Jun 21;19(6):e1004018. doi: 10.1371/journal.pmed.1004018 (PMC9212142; doi:10.1371/journal.pmed.1004018)
Supplement: Attachment — Submitted filename: PMEDICINE-D-21-05271R2 Rebuttal letter 20220429.docx [file pmed.1004018.s010.docx]

29 April 2022

Dr Caitlin Moyer

Associate Editor

PLOS Medicine

**RE: PMEDICINE-D-21-05271R2**

**Manuscript Title: Adverse events of special interest and mortality following vaccination with mRNA (BNT162b2) and inactivated (CoronaVac) SARS-CoV-2 vaccines in Hong Kong: A retrospective study**

Dear Dr Moyer,

On behalf of my co-authors, I would like to thank the editors and reviewers again for their valuable comments and suggestions. Responding to these, we have made the necessary adjustments in our revised manuscript, and hope that you will now find it suitable for publication. We provide our point-to-point responses to the earlier version of this paper reviewed.

**Requests from Editors:**

1. Title: Please use sentence capitalization for the title, and please update the title in both the manuscript submission system and the manuscript document. Please also revise the title to: “Adverse events of special interest and mortality following vaccination with mRNA (BNT162b2) and inactivated (CoronaVac) SARS-CoV-2 vaccines in Hong Kong: A retrospective study”

*Response: Thank you for the suggestion. We have now revised the title accordingly.*

2. Abstract: Methods and Findings: Lines 57-64: Please also report the associated p values, in addition to the 95% CIs, for comparisons between CoronaVac and BNT162b2.

*Response: We have now reported the associated p-values in addition to the 95%Cis for comparisons between CoronaVac and BNT162b2.*

*Relevant text in Abstract: “After the first dose, incidence rates of overall AESI (IRR=0.98, 95%CI=0.89–1.08, p=0.703) and mortality (IRR=0.96, 95%CI=0.63–1.48, p=0.868) associated with CoronaVac were generally comparable to BNT162b2, except for Bell’s palsy (IRR=1.95, 95%CI=1.12–3.41, p=0.018), anaphylaxis (IRR=0.34, 95%CI=0.14–0.79, p=0.012), and sleeping disturbance or disorder (IRR=0.66, 95%CI=0.49–0.89, p=0.006). After the second dose, incidence rates of overall AESI (IRR=0.97, 95%CI=0.87–1.08, p=0.545) and mortality (IRR=0.85, 95%CI=0.51–1.40, p=0.516) were comparable between CoronaVac and BNT162b2 recipients, with no significant differences observed for specific AESI.” (page 3 lines 57-65)*

3. Abstract: Methods and Findings: Please move the study limitations sentence to the end of the Methods and Findings paragraph, deleting the break at line 65.

*Response: We have now moved the sentence about study limitations to the end of the Methods and Findings paragraph (page 3 lines 65-67).*

4. Abstract: Line 77: Please use “low and middle income countries” instead of “developing countries” if this is what is meant.

*Response: Thank you for the suggestion. We have now replaced “developing countries” with “low and middle income countries.”*

*Relevant text in Abstract: “Our results could help inform the choice of inactivated COVID-19 vaccines mainly administered in low and middle income countries with large populations, in comparison to the safety of mRNA vaccines.” (pages 3-4 lines 74-77)*

5. Line 119: Please remove “Manuscript Text” from the document.

*Response: “Manuscript Text” is now removed from the document.*

6. Results: Line 362: Please re-word this as “AESI and all-cause mortality reported after the first dose of CoronaVac were compared to BNT162b2 as a reference group (Table 3).” if this is accurate.

*Response: Thank you for the suggestion. We have now revised the sentence accordingly.*

*Relevant text in Results: “AESI and all-cause mortality reported after the first dose of CoronaVac were compared to BNT162b2 as a reference group (Table 3).” (page 17 lines 360-361)*

7. Discussion: Line 443: We suggest “ in view of the multimorbidity status and relatively older ages of the deceased” if this is accurate.

*Response: Thank you for the suggestion. We have now revised the sentence accordingly.*

*Relevant text in Discussion: “Nevertheless, autopsy and postmortem findings have not contributed to establishing a causal link between COVID-19 vaccination and death, in view of the multimorbidity status and relatively older ages of the deceased, where decompensation could result from common side effects to immunization [63].” (page 20 lines 439-442)*

8. Discussion: Line 476: We suggest that “younger” is not needed in the sentence “...especially among younger males under the age of 30…”

*Response: Thank you for the suggestion. The word “younger” is now removed.*

*Relevant text in Discussion: “Since the rollout of massive vaccination and continuous safety monitoring, numerous studies have concluded that BNT162b2 is potentially associated with an increased risk of myocarditis, especially among males under the age of 30, and within the first week following second dose vaccination [14-16,69].” (pages 21-22 lines 473-476)*

9. Discussion: Line 538: Please use “low and middle income countries” instead of “developing countries” if this is what is meant.

*Response: Thank you for the suggestion. We have now replaced “developing countries” with “low and middle income countries.”*

*Relevant text in Discussion: “By evaluating and comparing the safety profile of CoronaVac against BNT162b2 (Comirnaty), the first FDA approved SARS-CoV-2 vaccine for COVID-19 prevention, this study could help inform the choice of inactivated COVID-19 vaccines mainly administered in low and middle income countries with large populations, in comparison to the safety of mRNA vaccines.” (page 24 lines 534-538)*

10. Line 562: Please remove the Ethical Approval description from this location. Please ensure that the study approvals, information on data anonymization, and participant consent waiver are completely described in the relevant place in the Methods (line 216).

*Response: The Ethical approval description is now removed from the Declarations section. Relevant information has been elaborated under Methods.*

*Relevant text in Methods: “Ethical approval for this study was granted by the Institutional Review Board of the University of Hong Kong/Hospital Authority Hong Kong West Cluster (UW 21-149 and UW 21-138); and the Department of Health Ethics Committee (LM 21/2021). A waiver of participant consent was granted for this retrospective cohort study using anonymized data.” (page 11 lines 215-218)*

11. Line 568: Availability of data and materials: Please remove this section from this location in the text. Please ensure that this information is completely and accurately entered into the data availability section of the manuscript submission metadata. In the event the article is accepted for publication, this information will be included.

*Response: The description of Availability of data and materials is now removed from the Declarations section. This information is now entered into the data availability section of the manuscript submission metadata.*

12. Line 581: Competing Interests: Please remove this section from this location in the text. Please ensure that this information is completely and accurately entered into the competing interests section of the manuscript submission system. In the event the article is accepted for publication, this information will be included.

*Response: The description of Competing interests is now removed from the Declarations section. This information is now entered into the competing interests section of the manuscript submission system.*

13. Line 609: Funding: Please remove this section from this location in the text. Please ensure that this information is completely and accurately entered into the funding section of the manuscript submission system. In the event the article is accepted for publication, this information will be included.

*Response: The description of Funding is now removed from the Declarations section. This information is now entered into the funding section of the manuscript submission system.*

14. Line 615: Author contributions: Please remove this section from this location in the text. Please ensure that this information is completely and accurately entered into the author contributions section of the manuscript submission system. In the event the article is accepted for publication, this information will be included.

*Response: The description of Author contributions is now removed from the Declarations section. This information is now entered into the author contributions section of the manuscript submission system.*

15. References: Please check each reference for formatting, including the NLM abbreviations for journal titles. Please use the "Vancouver" style for reference formatting, and see our website for other reference guidelines <https://journals.plos.org/plosmedicine/s/submission-guidelines#loc-references>

Please update references 45, 50,69, 70 with complete information.

*Response: The reference list is now reviewed and updated.*

16. S1 Figure: Please define IPTW in the legend.

*Response: The abbreviation IPTW (inverse probability of treatment weighting) is now elaborated in the caption of S1 Figure under Supporting Information (page 41). It is also added to S1 Fig in the legend.*

**Comments from the reviewers:**

**Reviewer #1:** The authors answered all queries. I have no further comment

*Response: Thank you very much for the comments on our paper.*

**Reviewer #2:** Thanks for the revised manuscript and responses to my initial queries. This is an excellent study and I recommend it should be published. Just one small change to the abstract (below).

The extra information about the study plan makes sense - that this is part of a wider surveillance study. There is clearly common support - this could be used as an example in teaching of appropriate amount of overlap of the PS. The weights looks reasonable with the trimming applied - no extreme weights indicating issues with PS estimation. The Poisson distribution clearly fits well from the extra info in S2_table.

*Response: Thank you very much for the comments and positive feedback.*

L67. I would just clarify slightly "..possibility of being underpowered for some AESI with very low observed incidences" as for many of the important AESI you are very well powered!

*Response: Thank you for the suggestion. We have revised the sentence accordingly.*

*Relevant text in Abstract: “Main limitations of this study include residual confounding due to its observational nature, and the possibility of being underpowered for some AESI with very low observed incidences.” (page 3 lines 65-67)*

We sincerely hope that you will consider this revised manuscript favorably. Should there be further corrections or clarifications needed, we are more than happy to make the necessary changes and provide additional information. We look forward to hearing from you soon.

Yours sincerely,

Ian Chi Kei Wong, PhD

Centre for Safe Medication Practice and Research, Department of Pharmacology and Pharmacy, LKS Faculty of Medicine, The University of Hong Kong

2/F, 21 Sassoon Road, Li Ka Shing Faculty of Medicine, Laboratory Block, Faculty of Medicine Building, Hong Kong SAR, China

Phone: (+852) 3917 9024

Fax: (+852) 2817 0859

Email: [wongick@hku.hk](mailto:wongick@hku.hk)
